# TDAG51 induces renal interstitial fibrosis through modulation of TGF-β receptor 1 in chronic kidney disease

**DOI:** 10.1038/s41419-021-04197-3

**Published:** 2021-10-08

**Authors:** Rachel E. Carlisle, Zahraa Mohammed-Ali, Chao Lu, Tamana Yousof, Victor Tat, Samera Nademi, Melissa E. MacDonald, Richard C. Austin, Jeffrey G. Dickhout

**Affiliations:** McMaster University and The Research Institute of St. Joe’s Hamilton, Department of Medicine, Division of Nephrology, Hamilton, Canada

**Keywords:** Endoplasmic reticulum, Apoptosis, End-stage renal disease, Preclinical research, Chronic inflammation

## Abstract

Chronic kidney disease (CKD) is characterized by the gradual loss of renal function and is a major public health concern. Risk factors for CKD include hypertension and proteinuria, both of which are associated with endoplasmic reticulum (ER) stress. ER stress-induced TDAG51 protein expression is increased at an early time point in mice with CKD. Based on these findings, wild-type and TDAG51 knock-out (TDKO) mice were used in an angiotensin II/deoxycorticosterone acetate/salt model of CKD. Both wild-type and TDKO mice developed hypertension, increased proteinuria and albuminuria, glomerular injury, and tubular damage. However, TDKO mice were protected from apoptosis and renal interstitial fibrosis. Human proximal tubular cells were used to demonstrate that TDAG51 expression induces apoptosis through a CHOP-dependent mechanism. Further, a mouse model of intrinsic acute kidney injury demonstrated that CHOP is required for ER stress-mediated apoptosis. Renal fibroblasts were used to demonstrate that TGF-β induces collagen production through an IRE1-dependent mechanism; cells treated with a TGF-β receptor 1 inhibitor prevented XBP1 splicing, a downstream consequence of IRE1 activation. Interestingly, TDKO mice express significantly less TGF-β receptor 1, thus, preventing TGF-β-mediated XBP1 splicing. In conclusion, TDAG51 induces apoptosis in the kidney through a CHOP-dependent mechanism, while contributing to renal interstitial fibrosis through a TGF-β-IRE1-XBP1 pathway.

## Introduction

Chronic kidney disease (CKD) is an increasingly prevalent health concern, with an incidence over 15% in the United States and Canada [1]. Hypertension and proteinuria are two common risk factors for the development of CKD and decline in renal function [2, 3]. Individuals with CKD develop reduced filtration capacity due to renal damage, including tubular atrophy and apoptosis, and renal interstitial fibrosis [3]. Both apoptosis and fibrosis have been associated with endoplasmic reticulum (ER) stress in the kidney [4, 5]; while the relationship between apoptosis and ER stress is well established, the mechanisms connecting ER stress and fibrosis are less clear.

ER stress develops when unfolded proteins accumulate in the ER, thereby activating the unfolded protein response (UPR). The UPR is an evolutionarily conserved cellular response primarily regulated by the ER-resident chaperone GRP78 (glucose-regulated protein 78). Misfolded proteins cause GRP78 to dissociate from the ER transmembrane proteins, PERK (PKR-like endoplasmic reticulum kinase), IRE1 (inositol-required enzyme 1), and ATF6 (activating transcription factor 6), thereby activating the UPR pathways. The UPR is considered a cell survival response, with the activated signaling pathways attempting to maintain proteostasis by reducing general protein translation and increasing the production of molecular chaperones. However, prolonged or severe activation of the UPR can lead to cell death [6].

Apoptosis plays a significant role in the renal injury of various etiologies, including both acute kidney injury (AKI) [7] and CKD [8, 9]. C/EBP homologous protein (CHOP) plays an important role in the induction of apoptosis, and is induced by the UPR. CHOP is comprised of two functional domains; one contains a DNA-binding domain and dimerization domain, while the other is responsible for altering gene transcription and inducing apoptosis [10]. Lack of CHOP is protective against both apoptosis and fibrosis in various animal models of disease [11, 12]. Increased levels of apoptosis are found in nearly all types of fibrosis, and cells undergoing apoptosis have both direct (paracrine signaling) and indirect (immune response modulation) effects on the development of fibrosis [13].

Renal interstitial fibrosis is caused by an accumulation of extracellular matrix components in the renal interstitium, and is a major factor in the progression of CKD. The cytokine TGF-β (transforming growth factor-β) is the primary mediator of fibrosis and is up-regulated in most forms of CKD [14]. TGF-β is secreted in an inactive form with TGF-β signaling initiated by its binding to TGF-β receptors 1 and 2. TGF-β receptors are homodimers situated in the plasma membrane that work together to induce signaling downstream of TGF-β [15].

The expression of T-cell death-associated gene 51 (TDAG51), also known as PHLDA1 (pleckstrin homology-like domain, family A member 1), is induced by numerous ER stress agents/conditions [16, 17]. Enhanced TDAG51 expression can lead to cell shape changes and detachment-mediated apoptosis in human vascular endothelial cells [17], as well as human proximal tubular cells [16]. However, the role of TDAG51 in the progression of CKD is not yet known. We hypothesized that TDAG51 induces apoptosis via CHOP induction and renal interstitial fibrosis through modulation of TGF-β1 receptor 1 expression. As such, a well-established mouse model of CKD was used in wild-type and TDAG51 knock out (TDKO) mice to study the effects of TDAG51 on CKD development.

## Methods

### Animal studies

Wild-type C57BL/6 (WT), TDKO, and CHOP knock out (CKO) male mice were used in this study; knock out mice were bred on a C57BL/6 background. WT and TDKO mice were bred in-house (backcrossed >9 times) and genotyped before being allocated to the appropriate treatment group. CKO mice were purchased from Jackson Laboratories (stock #: 005530). Mice were maintained in the St Joseph’s Animal Facility at McMaster University with free access to food and drinking water, and were housed with a 12-h light/dark cycle. Unfasted anaesthetized animals were sacrificed via exsanguination using cardiac puncture to collect blood; kidneys were harvested and fixed in 4% paraformaldehyde or frozen for biochemical analysis. The investigators were blinded to the groups when assessing outcomes by using non-descriptive codes to identify the samples. In all sample analyses, biological replicates were used to produce the mean result and SEM. All animal work was done in accordance with, and approved by, the McMaster University Animal Research Ethics Board.

#### Model of CKD

**Fig. 1:**
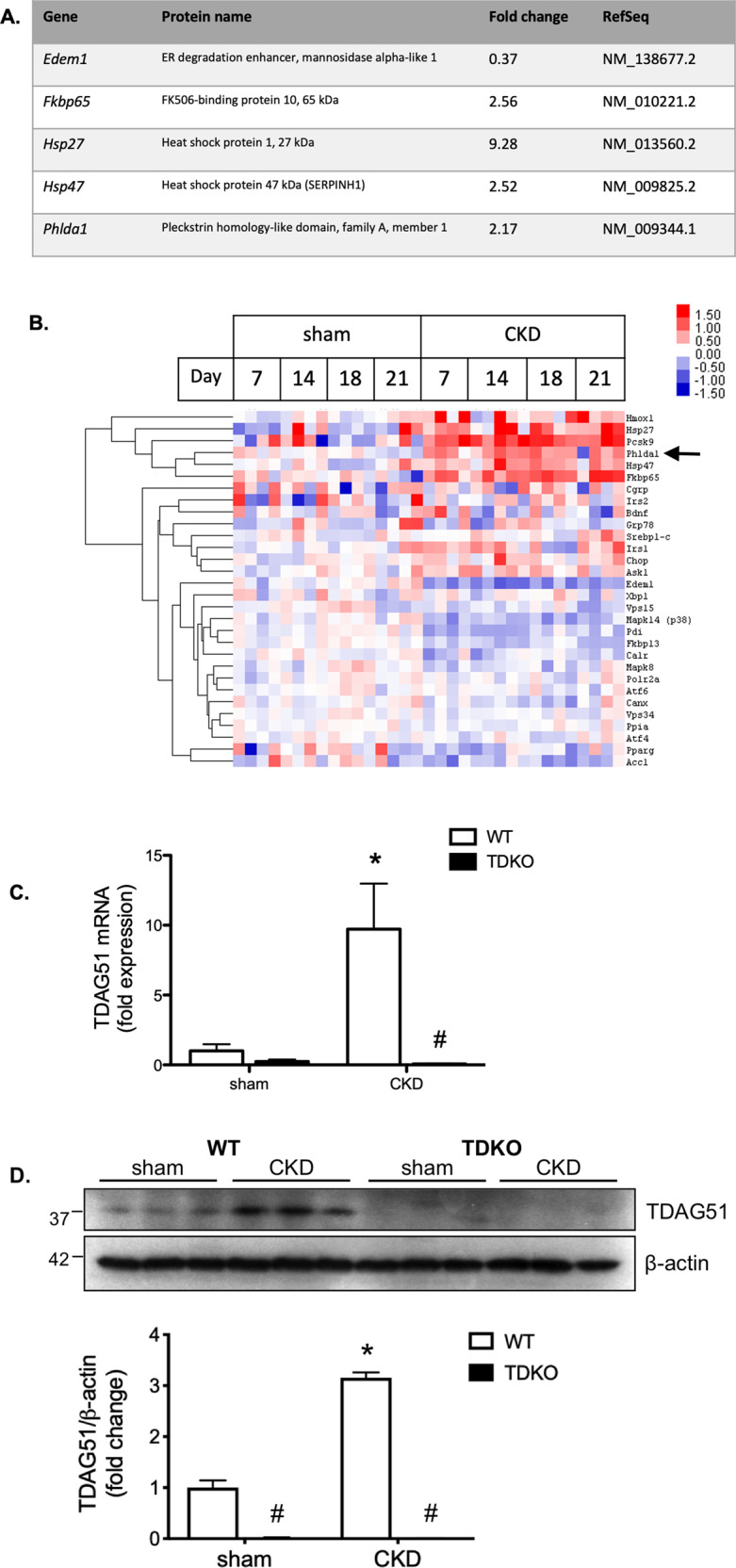
Renal injury increases TDAG51 expression. Renal tissue from WT and TDKO mice with chronic kidney disease (CKD) was analyzed to examine expression of TDAG51. **A** Microarray analysis demonstrates the gene for TDAG51, *Phlda1*, is upregulated in WT mice with CKD. **B** Nanostring analysis demonstrates that TDAG51 mRNA expression is elevated throughout the progression of CKD in mice (day 7, 14, 18, 21). **C** TDAG51 mRNA expression, as measured with RT-PCR, is increased in WT mice with CKD, but not found in TDKO mice. **D** WT mice with CKD have significantly more TDAG51 protein expression compared with sham WT mice. TDKO mice do not express any TDAG51 protein. *P < 0.05 vs sham; #, *P* < 0.05 vs WT.

Two weeks prior to the start of the experiment, 10-week-old mice were uninephrectomized. Animals were 12 weeks old at the start of the experiment. Mice were randomly allocated to one of two groups alternating based on the order of inclusion in the study: (1) sham: sham-operated, normal drinking water (WT, *n* = 9; TDKO, *n* = 8); or (2) CKD: subcutaneously implanted with an angiotensin II (1.5 ng/min/g; Sigma-Aldrich; Oakville, Canada) infusion pump (ALZET Osmotic Pumps; Cupterino, CA, USA) and deoxycorticosterone acetate (DOCA) pellet (Innovative Research of America; Sarasota, FL, USA), with 1% NaCl drinking water (WT, *n* = 9; TDKO, *n* = 8), as described previously [9]. Sample sizes were established based on previous work with the animal model to statistically determine a difference in the parameters measured. For nanostring analysis only (Fig. 1B), additional WT animals were sacrificed at days 7 (*n* = 4), 14 (*n* = 5), 18 (*n* = 4), and 21. TDKO mice were sacrificed on day 21. Prior to sacrifice, mice were placed in metabolic cages for 24 h for urine collection, and blood pressure was measured via tail-cuff (Kent Scientific; Torrington, CT, USA). No animals were excluded from the analysis.

#### Tunicamycin model of AKI

WT, TDKO, and CKO mice were randomly allocated to one of two groups alternating based on order of inclusion in the study: (1) sham: administered saline via intraperitoneal injection (WT, *n* = 6; TDKO, *n* = 4; CKO, *n* = 4); or (2) TM: administered tunicamycin (TM; 0.5 mg/kg; Sigma Aldrich) via intraperitoneal injection (WT, *n* = 6; TDKO, *n* = 6; CKO, *n* = 6). One additional group of WT mice was used (TM + 4-PBA). Mice were given 4-phenylbutyrate (4-PBA; 1 g/kg/day) (Scandinavian Formulas; Sellersville, PA, USA) in the drinking water from 7 days prior to TM injection, until sacrifice (*n* = 6). 72 h after injection, animals were sacrificed.

### Analysis of serum creatinine

Serum creatinine from WT and TDKO mice was analyzed using an enzymatic assay (Pointe Scientific; Mississauga, Canada) to confirm AKI according to KDIGO guidelines [18]. Analysis was performed following the manufacturer’s instructions. Briefly, a plasma sample from each mouse was incubated with ‘reagent 1’ in a 96-well plate at 37 °C for 5 min. The absorbance was read at 550 nm (A1) before ‘reagent 2’ was added and the plate was incubated for an additional 5 min at 37 °C. The absorbance was read once again (A2), and the change in the absorbance values (A2-A1) was interpolated into the standard curve, providing the creatinine value for each sample.

### DNA genotyping for wild type and mutant TDAG51

DNA genotyping was performed on tissue from WT and TDKO mice to confirm genetic strain, as previously published [19, 20]. Ear or tail tissue from each mouse was digested in digest buffer mix (97.5 μl DirectPCR (#101-T; Qiagen; Toronto, Canada) + 2.5 μl Proteinase K (#E00491; ThermoFisher; Mississauga, Canada)) at 60 °C for 16 h followed by 94 °C for 15 min. Two reactions were performed to confirm tissue genotype via PCR, one containing two wild-type TDAG51 primers and the other containing two mutant TDAG51 primers. Wild-type TDAG51 primers were: WT1: CCG CAG CAC CTC CAA CTC TGC CTG and WT2: GTC TTC AAA TAC AAT GAA AGA GTC. TDAG51 mutant primers were: KO1: AGA GCA GCC GAT TGT CTG TTG TGC CCA GTC, and KO2: AAA TGG AAG TAG CAC GTC TCA CTA GTC TCG. PCR was performed using one denaturation cycle (94 °C for 3 min), followed by 36 cycles of 94 °C for 30 s, 58 °C for 1 min, 72 °C for 1 min. A 1000 bp band was found in samples from wild-type mice, while a 400 bp band was found in samples from TDKO mice. TDAG51 heterozygous mice had both wild-type and mutant TDAG51 bands and were excluded from this study.

### RNA isolation and analysis

Total RNA was isolated from flash-frozen mouse kidney tissue using the RNeasy Mini Kit (Qiagen), as per the manufacturer’s instructions.

#### NanoString

RNA integrity was determined using the Agilent 2100 bioanalyzer and Agilent RNA 6000 Nano reagents (Agilent Technologies; Mississauga, Canada). Samples with an integrity number over 5 were used. Nanostring analysis was performed as previously [11]. A heat map was created with each box representing fold change of a gene (row) for a single animal with CKD compared to sham (column). Fold changes were log2-transformed. Downregulation is designated by blue, and upregulation is designated by red.

#### Rt-PCR

DNase digestion was performed as per the manufacturer’s instructions using recombinant DNase I, RNase-free (Roche; Mississauga, Canada), in order to eliminate DNA contamination. cDNA was synthesized from RNA using a High Capacity cDNA Reverse Transcription Kit (Applied Biosystems; Burlington, Canada) and the REVTRANS program on the Mastercycler gradient (Eppendorf; Mississauga, Canada). Relative levels of TDAG51, CHOP, sXBP1, and 18S were detected with Fast SYBR Green Master Mix (Applied Biosystems), and RT-PCR analysis was performed using 7500 Software (Applied Biosystems). Mouse TDAG51 primers were obtained as a primer mix (Qiagen). Sequences for all other primers used are as follows: for mouse CHOP forward: 5′–CTGCCTTTCACCTTGGAGAC–3′ and reverse: 5′– CGTTTCCTGGGGATGAGATA–3′. For mouse sXBP1 forward: 5′– TCCGCAGCAGGTGCAGG–3′ and reverse: 5′–GCCCAAAAGGATATCAGACTCAGA–3′. For mouse 18S forward: 5′– AGTCCCTGCCCTTTGTACACA–3′ and reverse: 5′– CGATCCGAGGGCCTCACT–3′. For rat sXBP1 forward: 5′– CTGAGTCCGAATCAGGTGCAG–3′ and reverse: 5′– ATCCATGGGAAGATGTTCTGG–3′. For rat 18S forward: 5′– GTTGGTTTTCGGAACTGAGGC–3′ and reverse: 5′– GTCGGCATCGTTTATGGTCG–3′.

#### PCR

PCR was conducted on HK-2 cells for sXBP1 and 18S. RNA was reverse transcribed using the High-Capacity cDNA Reverse Transcription Kit (Applied Biosystems), as per the manufacturer’s directions. cDNA was amplified with recombinant *Taq* polymerase (Invitrogen; Mississauga, Canada). PCR products were digested with *Pst*I for 1 h at 37 °C, separated on a 2% agarose gel, and visualized using Safe-Red (Applied Biological Materials Inc; Burlington, Canada) and the ChemiDoc XRS + system (BioRad; Mississauga, Canada).

### Western blotting

Renal cortical tissue (CKD groups), pars recta (TM groups), or human proximal tubular epithelial cells were lysed in 4X SDS lysis buffer containing protease and phosphatase inhibitors. Lysates underwent a BioRad DC protein assay to determine protein levels, and subsequently were separated in a 10% SDS-PAGE reducing gel, and transferred to a nitrocellulose membrane. Antibodies to TDAG51 (sc-23866; Santa Cruz Biotechnology; Santa Cruz, CA, USA) and CHOP (sc-7351; Santa Cruz) were diluted 1:200, KDEL (SPA-827; Stressgen; Burlington, Canada) was diluted 1:1000 (used against the KDEL amino acid sequence of GRP78), TGFβ-R1 (MAB587; R&D systems; Oakville, Canada) was diluted 1:500, and β-actin (66009-1; ProteinTech; Rosemont, IL, USA) was diluted 1:5000. Appropriate horseradish peroxidase-conjugated secondary antibodies (1:5000; goat anti-mouse; BioRad) and ECL Western blotting detection reagents were used to detect specific proteins. Band intensities were densitometrically quantified using ImageLab software and expressed as a ratio of β-actin loading control.

### Urinalysis

Prior to sacrifice, animals were placed in metabolic cages for 24 h. Food and water intake were recorded, and urine was collected. Our in-house laboratory (St Joseph’s Healthcare Hamilton) was responsible for measuring levels of total urinary protein. Albuminuria was measured via ELISA (Bethyl Laboratories Inc; Burlington, Canada).

### Tissue staining and quantification

A microtome was used to cut formalin-fixed paraffin-embedded kidney tissue blocks into sections (4 μm thick). Images of histological or immunohistochemical staining were taken using a BX41 Olympus microscope. Images of fluorescent staining were taken using an Olympus IX81 Nipkow scanning disc confocal microscope.

#### Periodic acid-Schiff

Periodic acid-Schiff staining was performed as previously [7]. Briefly, slides were oxidized in 1% aqueous periodic acid (Sigma Aldrich), rinsed, treated with Schiff reagent (Electron Microscopy Sciences; Hatfield, PA, USA), washed, and stained with haematoxylin (Sigma Aldrich). Glomeruli were quantified as described previously [21]. Protein casts were quantified using Metamorph image analysis software. Briefly, casts were outlined by hand, and the area of each cast was recorded. Protein cast areas were summed in each image to determine the area density of protein cast deposition. Protein casts in the cortex and medulla were analyzed separately. Tubular dilation was quantified in a similar manner using ImageJ software. The tubule lumen, the area inside the tubule lining, was outlined by hand, and the area of each lumen in each image was recorded. The lumen areas for each image were summed and a percentage of lumen area/total image area was calculated and averaged for each animal. Lumen areas from the cortex and medulla were analyzed separately.

#### TUNEL

Immunohistochemical staining was performed using TACS 2 Tdt-Fluor In Situ Apoptosis Detection Kit (Trevigen; Burlington, Canada). Briefly, after sections were de-paraffinized, they were incubated with Proteinase K (1:50 in ddH_2_O) for 30 min at 37 °C. Endogenous peroxidase quenching was performed for 5 min. Slides were coated in Labeling Reaction Mix at 37 °C for 1 h and immersed in 1× TdT Stop Buffer to stop the labeling reaction. Slides were then incubated with horseradish peroxidase-streptavidin (Vector Laboratories; Burlington, Canada), followed by Nova Red (Vector Laboratories) and haematoxylin (Sigma Aldrich). Immunofluorescent TUNEL staining was performed as previously [7], using a TMR-in situ cell death detection kit (Roche).

#### Immunohistochemical staining

Staining for α-smooth muscle actin, fibronectin, and cleaved caspase 3 was performed as previously [11]. Slides were incubated with endogenous peroxidase, underwent heat-induced antigen retrieval with citric acid (fibronectin and cleaved caspase 3), and blocked with normal goat serum. Primary antibodies were incubated overnight at 4 °C. Sections were subsequently incubated with biotinylated secondary antibodies, streptavidin/peroxidase, and Nova Red. Antibodies for α-smooth muscle actin (Sigma Aldrich; A2547) were diluted at 1:200, for fibronectin (Sigma Aldrich; F3648) were diluted at 1:200, and for cleaved caspase 3 (Cell Signaling; #9661) were diluted at 1:400.

#### Masson Trichrome staining

Masson Trichrome staining was performed as per the manufacturer’s instructions (Sigma Aldrich). Briefly, after de-paraffinization, sections were incubated overnight in Zenker’s solution at room temperature. Sections were stained with haematoxylin, washed, and subsequently stained with Biebrich Scarlet-Acid Fuchsin solution, phosphotungstic/phosphomolybdic acid solution, and aniline blue solution. Slides were then washed in 1% acetic acid before dehydration and mounting. Collagen deposition is indicated by blue color.

#### Immunofluorescent CHOP staining

Sections were permeabilized with 0.1% triton-X, blocked with normal goat serum, and antigen retrieval was performed using citric acid buffer. Primary antibodies were applied at 1:40 (CHOP; sc-575; Santa Cruz). Appropriate fluorescently-tagged secondary antibodies (647 nm) were applied, followed by DAPI staining.

### Cell culture and chemicals

Immortalized human proximal tubular epithelial (HK-2; ATCC; Burlington, Canada; CRL-2190) cells and rat renal fibroblasts (Cell Biologics; Burlington, Canada; RA-6016) were grown in low glucose/F12 media (Gibco; Burlington, Canada) in 10% FBS (Gibco) with 1% penicillin/streptomycin (Gibco). Thapsigargin was used at 500 nM (Sigma Aldrich), L-ascorbic acid (AA2P) (Sigma Aldrich; A8960) at 25 μg/ml, transforming growth factor-β1 (TGF-β1) (R&D systems; #240-B) at 5 ng/ml, the IRE1 endonuclease inhibitor 4μ8C (Calbiochem; Oakville, Canada; #412512) at 30 μM, tunicamycin (Sigma Aldrich) at 1 μg/ml, and the TGFβ-R1 inhibitor SB431542 (Cayman Chemical Company; Ann Arbor, MI, USA; #13031) at 10 μM.

### Transfection and immunofluorescent staining in vitro

HK-2 cells were transfected with eGFP or TDAG51 bound to GFP (TD-GFP). 48 h after transfection, cells were fixed in 4% paraformaldehyde, and immunofluorescently stained with CHOP antibodies (red; sc-7351; Santa Cruz) and a fluorescently labeled secondary antibody. Cell nuclei were stained with DAPI (blue). HK-2 cells were also transfected with pcDNA3.1 control vector or pcDNA3.1 vector with human CHOP gene. 72 h after transfection, cells were fixed and immunofluorescently stained with TUNEL (red; Roche) for apoptosis and CHOP antibodies (green; sc-575; Santa Cruz) with a fluorescently labeled secondary antibody. Cells were imaged using an Olympus IX81 Nipkow scanning disc confocal microscope.

### Picrosirius red spectrophotometric assay

Collagen production was measured using a picrosirius red-based assay, as previously described [22]. Briefly, renal fibroblasts were treated with TGF-β1, AA2P, TGF-β1, and AA2P, or TGF-β1 and AA2P and 4μ8c. After 72 h, cells were fixed in methanol for 10 mins at -20 °C. Cells were subsequently washed and incubated with picrosirius red stain (0.1% Direct Red 80 in saturated picric acid; 1 h at room temperature). Cells were then washed 3× with 0.1% acetic acid and imaged using a BX41 Olympus light microscope. The dye was subsequently eluted from the cells with 0.1 N NaOH (10 min on a rocking platform) and moved to a 96-well plate. The absorbance of picrosirius red stain was measured using a Spectramax Plus Microplate Reader at 540 nm.

### Statistical analysis

Each experiment was independently performed at least three times. Statistical analysis was performed on biological replicates using GraphPad Prism software. Quantitative results were statistically analyzed using two-sided, unpaired Student’s *T*-test for comparisons between two means, or one-way ANOVA for comparisons between three or more means (using Tukey post-hoc test). Results are expressed as the mean ± standard error of the mean. The significance of differences in the means was determined at a level of less than 5% (*P* < 0.05) probability of the difference occurring by chance.

## Results

To establish that expression of TDAG51 is regulated by CKD, mRNA and protein levels from mouse kidneys were analyzed. Microarray analysis was performed on WT renal tissue from sham and CKD mice. A number of UPR genes were significantly altered, including *Phlda1*, the human homolog of *TDAG51* (Fig. 1A). NanoString analysis confirmed TDAG51 mRNA was increased in CKD as early as 7 days after initiation of the CKD model. Levels of mRNA remained consistently high throughout the 21 days of the model (Fig. 1B). Mice were genotyped to confirm WT or TDKO mouse strain (Supplemental Fig. 1). Analysis of mRNA also confirmed the knockout of TDAG51 in TDKO mice (Fig. 1C). Western blotting was performed on lysates from the renal cortex of WT or TDKO mice with or without CKD. CKD increased expression of TDAG51 in WT mice; as expected, TDKO mice with or without CKD did not express the TDAG51 protein (Fig. 1D).

**Fig. 2:**
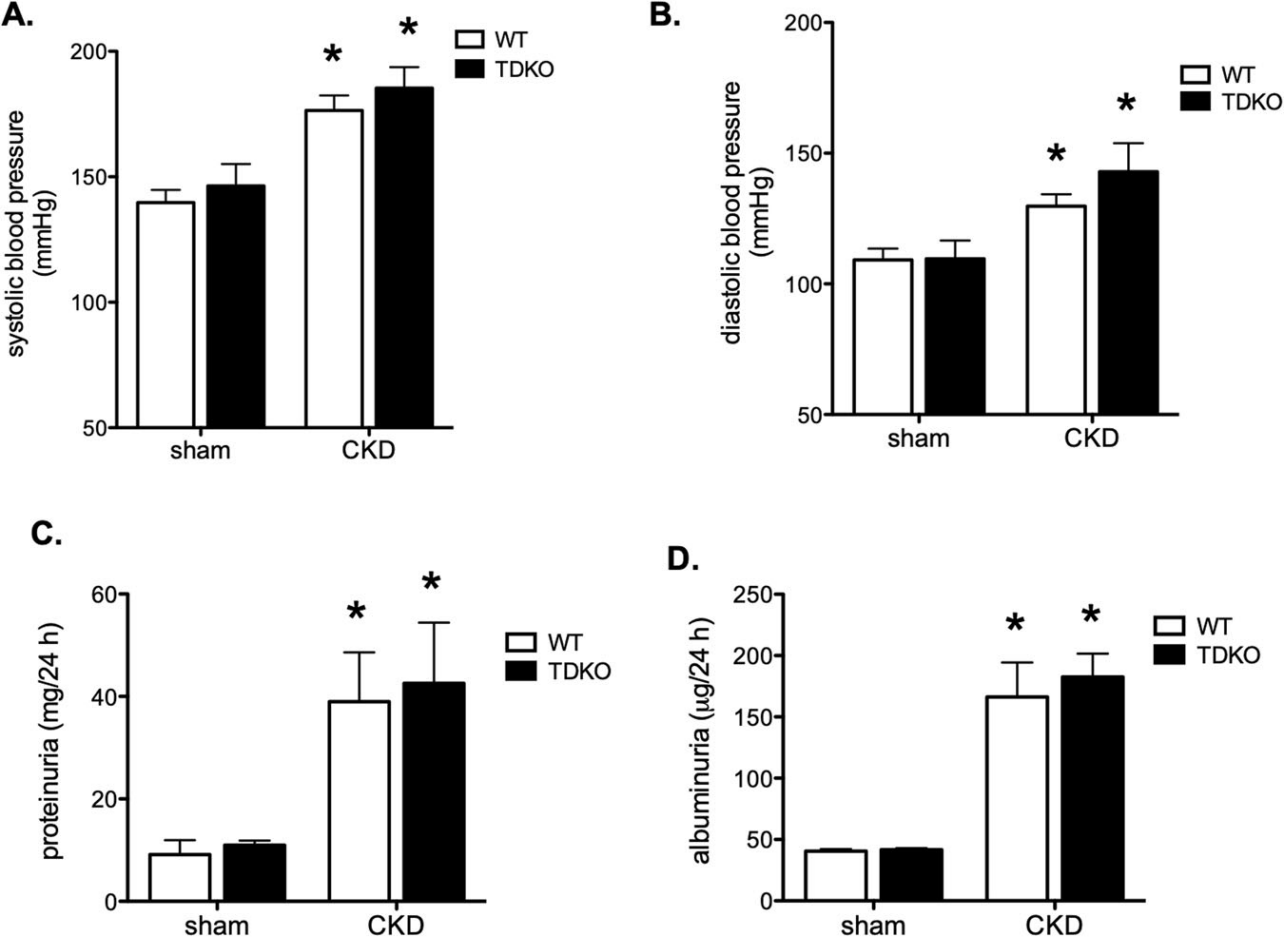
Knock out of TDAG51 does not protect against risk factors for kidney disease. Blood pressure was measured in WT and TDKO mice with or without chronic kidney disease (CKD). Systolic (**A**) and diastolic (**B**) blood pressures are increased in mice with CKD; no difference was found between WT and TDKO animals. Proteinuria (**C**) and albuminuria (**D**) are also increased in animals with CKD; knock out of TDAG51 had no effect on proteinuria or albuminuria levels. *P < 0.05 vs sham.

To examine the effect of TDAG51 absence on indicators of CKD progression, blood pressure, proteinuria, and albuminuria were measured. Systolic (Fig. 2A) and diastolic (Fig. 2B) blood pressures were increased in mice with CKD, but were not significantly different between WT and TDKO groups. Proteinuria (Fig. 2C) and albuminuria (Fig. 2D) were also increased in mice with CKD, but no differences were found between WT and TDKO groups.

**Fig. 3:**
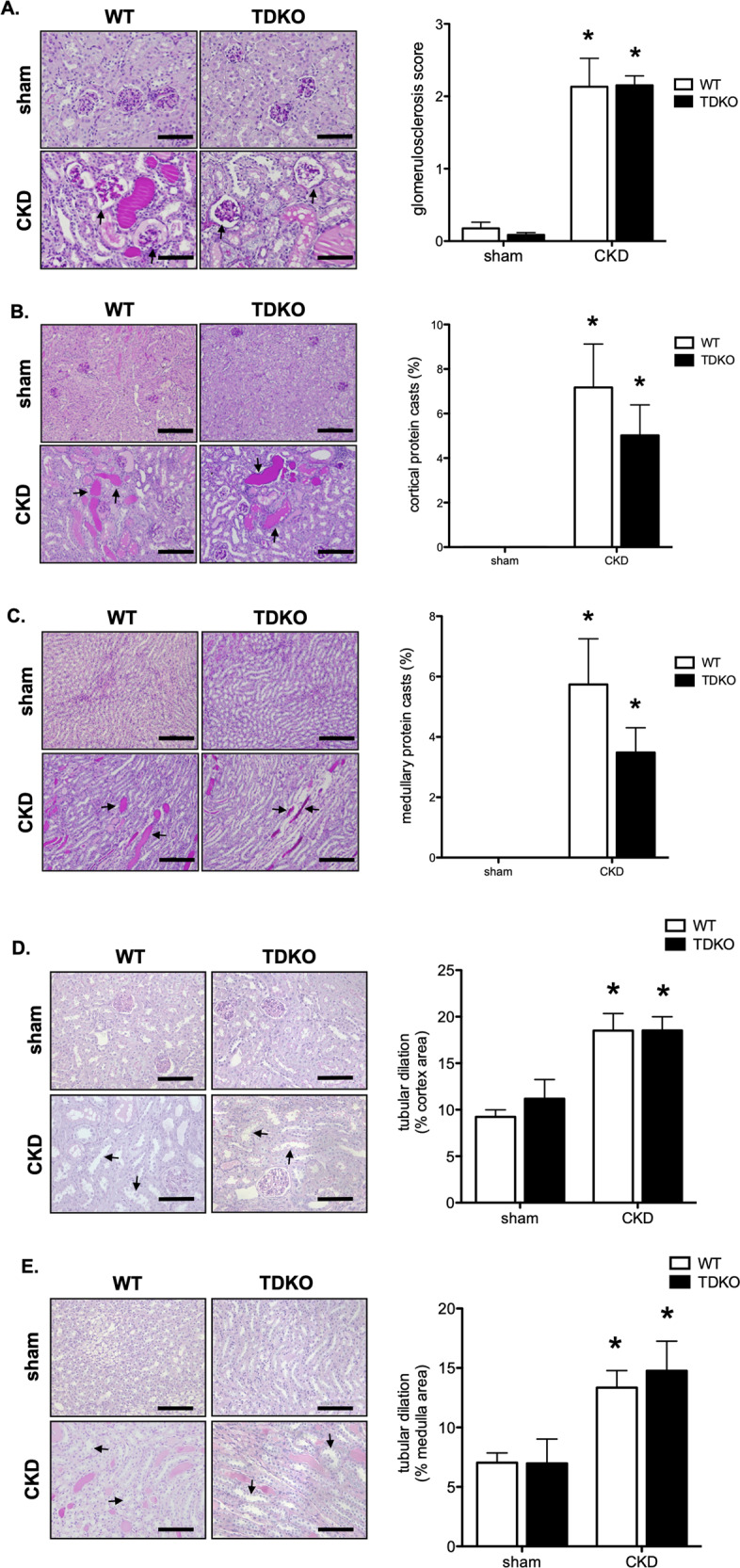
TDKO mice are not protected from glomerulosclerosis or tubular injury. Kidneys from WT and TDKO mice with or without chronic kidney disease (CKD) were examined for structural damage. **A** Glomerulosclerosis is increased in CKD animals, but there is no difference between WT and TDKO mice. Bar = 100 μm. Formation of protein casts is increased in the cortex (**B**) and medulla (**C**) of WT and TDKO mice with CKD. Tubular dilation was increased in the cortex (**D**) and medulla (**E**) of mice with CKD, and was not different between WT and TDKO mice. Bar = 200 μm. *P < 0.05 vs sham.

To determine if structural renal damage induced by this model was affected by the genetic knock out of TDAG51, kidneys were stained with PAS and analyzed. Glomerulosclerosis was increased in mice with CKD; however, glomerular damage was not significantly different between WT and TDKO mice (Fig. 3A). Protein cast deposition was analyzed in the cortex (Fig. 3B) and medulla (Fig. 3C) of the kidney. There was an increase in protein cast formation in mice with CKD, but no difference was found between WT and TDKO mice. Tubular dilation was also measured in the cortex (Fig. 3D) and medulla (Fig. 3E) of the kidney. While tubular dilation was increased with CKD, no difference was found between WT and TDKO mice.

**Fig. 4:**
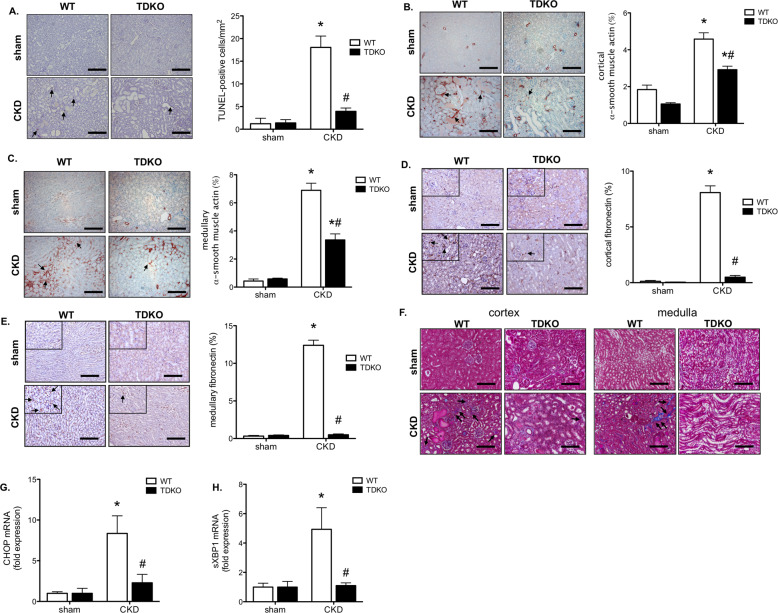
TDAG51 knock out prevents apoptosis and reduces renal interstitial fibrosis in chronic kidney disease. **A** Kidneys were stained with TUNEL to measure apoptotic cell death (arrows). WT mice with CKD demonstrated increased apoptosis in the cortex, primarily the proximal tubules, of the kidney. TDKO mice with CKD did not experience increased apoptosis. Kidneys were examined for markers of renal interstitial fibrosis. α-smooth muscle actin (SMA) is increased in the cortex (**B**) and medulla (**C**) of WT mice with CKD, when compared with sham WT mice. TDKO mice with CKD also demonstrated increased fibrosis in the kidney, though levels of α-SMA were significantly lower than WT mice. Fibronectin levels were increased in the (**D**) cortex and (**E**) medulla of WT mice with CKD; fibronectin expression was inhibited in TDKO mice with CKD. **F** Masson Trichrome stain reveals less collagen deposition (blue; arrows) in TDKO mice with CKD compared with WT mice with CKD. Renal tissue demonstrated increased CHOP mRNA (**G**) and sXBP1 (**H**) in WT mice with CKD, but not TDKO mice with CKD. Bar = 200 μm. *P < 0.05 vs sham; #, *P* < 0.05 vs WT.

Mouse kidneys were analyzed to investigate the role of TDAG51 in apoptosis and renal interstitial fibrosis in this model of CKD. The number of TUNEL-stained apoptotic cells was significantly increased in WT mice with CKD, but not TDKO mice with CKD. The location of the apoptotic cells indicates it was primarily proximal tubular epithelial cells in the cortex that were undergoing apoptosis in this model (Fig. 4A). Quantification of α-SMA staining demonstrates increased myofibroblast differentiation in the cortex (Fig. 4B) and medulla (Fig. 4C) of WT mice with CKD. Knock out of TDAG51 partially prevents the development and formation of α-SMA expressing cells. Similar results were found with fibronectin staining, which was increased in the cortex (Fig. 4D) and medulla (Fig. 4E) of WT CKD mice, but not in TDKO CKD mice. Collagen deposition (blue; arrows) is found in WT mice with CKD, with less deposition seen in TDKO mice with CKD (Fig. 4F). Gene expression of the pro-apoptotic protein CHOP was measured and found to be upregulated in WT mice with CKD, but not TDKO mice with CKD (Fig. 4G). Further, the activated form of XBP1 (spliced XBP1, sXBP1), a pro-fibrotic transcription factor, was increased in WT mice with CKD, but not TDKO mice with CKD (Fig. 4H).

**Fig. 5:**
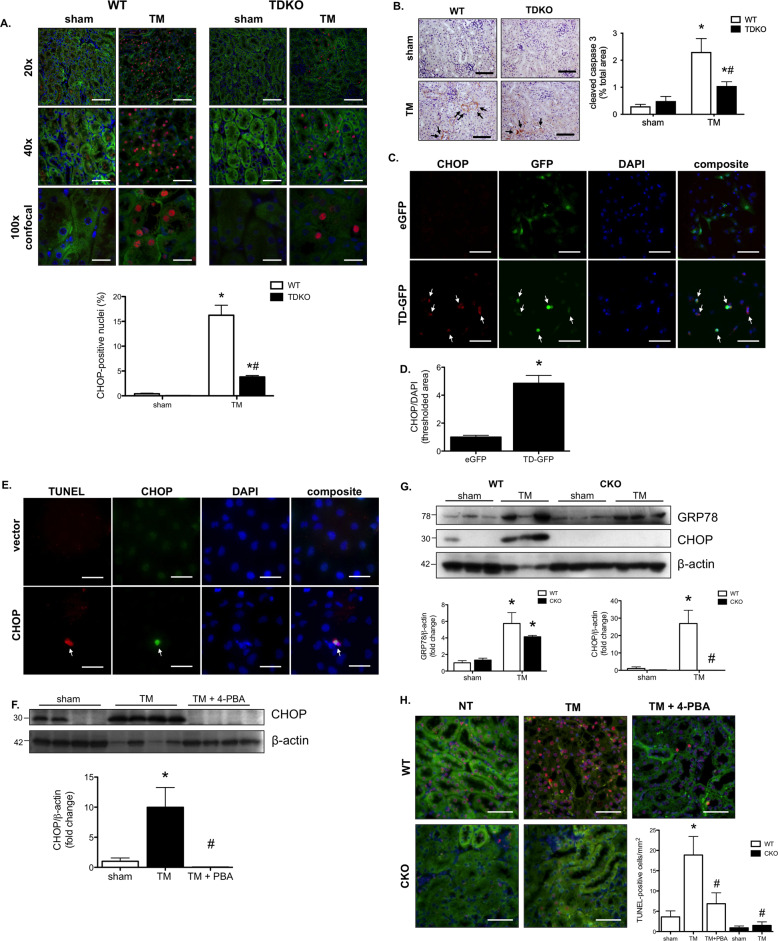
Reduced levels of CHOP prevent apoptosis in TDAG51 knock-out mice. **A** WT and TDKO mice were treated with tunicamycin (TM) for 72 h. CHOP expression (red) was significantly increased in WT mice treated with TM. TDKO mice treated with TM had significantly lower levels of CHOP compared with WT mice, though levels were not completely inhibited. 20× bar = 100 μm; 40× bar = 50 μm; 100× bar = 20 μm. **B** Cleaved caspase 3 was increased in the renal cortex of WT mice treated with TM and partially inhibited in TDKO mice treated with TM. Bar = 100 μm. **C** Human proximal tubular (HK-2) cells were transfected with eGFP or TDAG51-GFP (TD-GFP) for 48 h and stained for CHOP (red). Bar = 100 μm. **D** Cells transfected with TD-GFP expressed significantly more CHOP than eGFP-transfected cells. **E** HK-2 cells were transfected with a vector plasmid or with CHOP, and subsequently stained for CHOP (green) and apoptosis (TUNEL; red). Cells overexpressing CHOP are more highly associated with apoptosis. Bar = 20 μm. **F** WT mice were treated with TM with or without 4-phenylbutyrate (4-PBA). Western blotting demonstrates increased CHOP expression in kidneys from TM-treated WT mice. Co-treatment with 4-PBA prevented CHOP expression in the kidney. **G** WT and CKO mice were treated with TM for 72 h. WT and CKO mice demonstrate ER stress induction (via GRP78 expression) with TM treatment. CHOP expression is increased in WT mice, but absent in CKO mice. **H** Kidneys of TM-treated WT and CKO mice were stained for apoptosis (TUNEL; red). Quantification demonstrates that TM-induced apoptosis in WT mice, which was prevented with ER stress inhibition by 4-PBA. Knock out of CHOP also prevented TM-induced apoptosis in the kidney. Bar = 50 μm. *P < 0.05 vs sham; #, *P* < 0.05 vs WT TM.

To examine the role of TDAG51 on ER stress-induced kidney injury, a model of tunicamycin (TM)-mediated (and CHOP-dependent) AKI was used in WT and TDKO mice [7, 23]. TM was confirmed to cause stage 3 AKI in WT mice (serum creatinine >3× baseline) and stage 2 AKI in TDKO mice (serum creatinine 2.0–2.9× baseline), per the KDIGO guidelines [18] (Supplemental Fig. 2). The background emission of the tubular elements of the kidney was imaged with a FITC filter (green) allowing anatomical identification of renal structures. This allowed the identification of the structures where CHOP staining (red) occurred, which was primarily the proximal tubules of the pars recta. The pars recta are in the outer stripe of the medulla and are, incidentally, the region where most TM-mediated renal damage occurs. While TM-induced CHOP expression in WT mice, this increase in CHOP was partially inhibited in TDKO animals (Fig. 5A). Kidneys from WT and TDKO mice treated with TM were stained for cleaved caspase 3, a marker of apoptosis that is downstream of CHOP (arrows). WT mice exhibited increased staining for cleaved caspase 3 when treated with TM; TDAG51 deficiency was partially protective against elevated cleaved caspase 3 expression (Fig. 5B). To assess if TDAG51 had a direct effect on CHOP expression, human proximal tubular (HK-2) cells were transfected with either the TDAG51 plasmid vector (eGFP) or the vector containing the open reading frame for human TDAG51 (eGFP-TDAG51) for 48 h. Cells overexpressing eGFP or TDAG51 (bound to GFP; green) were stained for CHOP (red) (Fig. 5C). Transfection efficiency was similar between the two treatments (Supplemental Fig. 3). TDAG51-transfected cells had increased CHOP expression after 48 h, compared with eGFP-vector-transfected control cells (Fig. 5D). To assess the effect of CHOP overexpression directly inducing apoptosis, CHOP overexpression was performed in HK-2 cells. In this case, HK-2 cells overexpressing CHOP (green) co-localized with TUNEL-staining (red), demonstrating that increased CHOP expression promotes apoptosis (Fig. 5E). To examine this phenomenon in the whole kidney, mice were treated with TM to induce renal CHOP expression. These mice expressed significantly more CHOP in the renal cortex, which is inhibited with the protein-folding chaperone, 4-PBA (Fig. 5F). The UPR is activated (as demonstrated by increased GRP78 expression) in WT and CKO mice treated with TM; however, CHOP expression is absent in CKO mice (Fig. 5G). TM-treated WT mice demonstrate significantly more apoptosis (red), compared with sham mice. Co-treatment with the protein-folding chaperone, 4-PBA, prevents apoptosis from occurring in these mice. Further, CKO mice treated with TM exhibit almost no apoptosis (Fig. 5H).

**Fig. 6:**
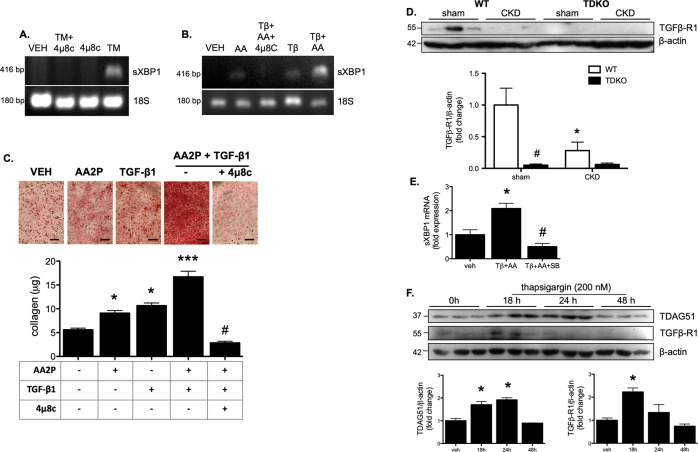
Renal interstitial fibrosis is mediated by sXBP1. **A** Rat renal fibroblasts were treated with TM and the IRE1 endonuclease inhibitor 4μ8c. TM-induced splicing of XBP1, which was prevented by 4μ8c treatment. **B** Renal fibroblasts were treated with TGF-β1 (Tβ) and ascorbic acid (AA2P; AA) to induce XBP1 splicing. Individually, TGF-β1 and AA2P increased XBP1 splicing, but the effect was increased with combined treatment. Co-treatment with 4μ8c was able to prevent TGF-β1-mediated XBP1 splicing. **C** Renal fibroblasts treated with TGF-β1 and AA2P show increased collagen deposition, which was inhibited with 4μ8c co-treatment. Bar = 100 μm. *P < 0.05 vs veh; ***P < 0.001 vs veh; #, *P* < 0.05 vs AA2P + TGF-β1. **D** Western blotting demonstrates that expression of TGF-β1 receptor (TGFβ-R1) is significantly reduced in kidneys from WT mice with CKD. Kidneys from TDKO mice with or without CKD do not express TGFβ-R1. *P < 0.05 vs sham; #, *P* < 0.05 vs WT. **E** Renal fibroblasts were treated with TGF-β1 and AA2P with or without SB431542 (SB), a TGFβ-R1 inhibitor. While TGF-β1 and AA2P increased mRNA levels of spliced XBP1, co-treatment with SB431542 inhibited XBP1 splicing. *P < 0.05 vs veh; #, *P* < 0.05 vs Tβ + AA. **F** HK-2 cells were treated with thapsigargin for varying time points (0, 18, 24, 48 h) and subsequently underwent western blotting. Thapsigargin induced TDAG51 expression at 18 and 24 h, but not 48 h. TGFβ-R1 was increased after 18 h of TG, but the expression was reduced at 24 and 48 h. *P < 0.05 vs 0 h.

To explore the role of TDAG51 in the development of renal interstitial fibrosis, rat renal fibroblasts were used. Fibroblasts were treated with TM for 6 h, which induced sXBP1. Co-treatment with 4μ8C, an IRE1 endonuclease inhibitor, prevented splicing of the transcription factor (Fig. 6A). Fibroblasts were then treated with TGF-β1 and L-ascorbic acid (AA2P) for 24 h, an essential cofactor for the enzymes involved in collagen biosynthesis, and XBP1 splicing was examined. Here, under these conditions that induce collagen biosynthesis, it was found that levels of sXBP1 were increased. Again, co-treatment with 4μ8C prevented XBP1 splicing (Fig. 6B). TGF-β and AA2P induced collagen production (red stain) in fibroblasts after 72 h, which 4μ8C was able to prevent (Fig. 6C). In order to examine how TDAG51 expression affects pro-fibrotic signaling, kidney lysates from WT and TDKO mice with or without CKD were probed for TGFβ-R1. Expression of the receptor decreased in WT mice with CKD. Sham-treated TDKO mice had significantly less TGFβ-R1 compared with WT mice, and levels did not change with CKD (Fig. 6D). Renal fibroblasts were treated for 24 h with TGF-β1 and AA2P with or without the TGFβ-R1 inhibitor SB431542. Treatment with TGF-β1 and AA2P induced splicing of XBP1, which was prevented with inhibition of TGFβ-R1 (Fig. 6E). HK-2 cells were treated with the ER stress inducer thapsigargin (TG; 500 M) for varying time points (0, 18, 24, 48 h) to induce TDAG51 and subsequently examine the expression levels of TGFβ-R1. TG treatment increased TDAG51 expression at 18 and 24 h, but not 48 h. TGFβ-R1 expression was increased at 18 h, but not 24 or 48 h (Fig. 6F).

## Discussion

Our mouse model of CKD altered the expression of a number of UPR genes, namely *Edem1*, *Fkbp65*, *Hsp27*, *Hsp47*, and *Phlda1*. While all of these genes play some role in the UPR, not all have fibrotic effects like those found in our model of CKD; further, the mechanisms and downstream effects of some are more understood than others. ER degradation enhancer, mannosidase α-like 1 (EDEM1) is a mannose-binding protein induced by ER stress, which triggers proteasomal degradation of misfolded glycoproteins in the ER [24]. Overexpression of IRE1 induces expression of EDEM1 [25], while silencing EDEM1 increases the bioavailability of ATF6 by increasing its stabilization [26]. FK506 binding protein 10 (65 kDa) (FKBP65) is an ER-resident molecular chaperone that has prolyl isomerase activity [27]. FKBP65 regulates the expression of α-smooth muscle actin [28] and collagen [29], and is induced by the pro-fibrotic cytokine TGF-β in pulmonary fibrosis [28]. Further, inhibiting FKBP65 prevents the development of bleomycin-induced idiopathic pulmonary fibrosis [28]. Heat shock proteins 27 (HSP27) and 47 (HSP47) are both involved in the development of renal interstitial fibrosis [30, 31]. The overexpression of HSP27 can be protective against fibrotic development [32], while inhibiting expression of the collagen-folding chaperone HSP47 prevents upregulation of TGF-β-induced fibrotic proteins [33]. While it is known that PHLDA1/TDAG51 is induced by the UPR and is necessary for the development of TGF-β adenovirus-mediated peritoneal fibrosis [16], the mechanism by which TDAG51 affects the progression of fibrosis, and specifically renal interstitial fibrosis in CKD, has not yet been studied.

Important risk factors for the progression of CKD include high blood pressure, proteinuria, and albuminuria. Hypertension is the leading risk factor for premature death worldwide, and is considered the most significant risk factor for CKD [2]; however, the most accurate predictor of CKD progression is proteinuria or albuminuria [2]. The glomerular injury allows proteins from the blood to filter through the glomerulus and infiltrate the urine, causing proteinuria and albuminuria. These important risk factors for CKD progression are not different between WT and TDKO mice, demonstrating TDAG51 expression does not influence blood pressure or proteinuria. Further, glomerular injury, protein cast formation, and tubular dilation are similar in WT and TDKO mice, suggesting these are not affected by TDAG51 either, which may represent upstream parameters that mediate TDAG51 expression.

TDAG51 is considered a translational regulator and is induced by various ER stress mediators, including thapsigargin and peroxynitrite [16, 34]. TDAG51 expression is mediated by the PERK and IRE1 arms of the UPR [35]. Upregulation of TDAG51 can lead to cell shape change and decreased cell adhesion, resulting in detachment-mediated apoptosis in human vascular endothelial cells [17] and human proximal tubular epithelial cells [16]. TDAG51 contains a pleckstrin homology-like domain, which plays a role in cytoskeletal organization [36]; it also contains proline-glutamine and proline-histidine repeats, which are typically found in proteins responsible for transcriptional regulation and/or apoptosis [37, 38].

As mentioned, glomerular injury can cause albuminuria, and can also lead to protein cast formation. Glomerular injury is associated with hyperfiltration of the nephron, whereby podocytes may be damaged and no longer provide a barrier to prevent plasma proteins from leaving the blood upon filtration. Reduced nephron numbers, such as that caused by uninephrectomy, triggers compensatory hyperfiltration, which in turn damages the podocytes and impairs the filtration barrier [39, 40]. The ablation of TDAG51 did not affect glomerular injury or its downstream effects (albuminuria, protein cast formation). However, CKO mice on our CKD model are protected from renal damage, including glomerular injury [11], and our current work suggests the pro-apoptotic protein CHOP is downstream of TDAG51. As such, it appears that glomerular injury is independent from TDAG51 influence but is dependent on a different mechanism that induces CHOP.

CHOP, also known as GADD153 or DDIT3, is known to play a role in apoptosis and is typically absent or expressed at very low levels in healthy cells [23]. When discovered, it was believed that CHOP expression was only increased in response to DNA damage; however, a number of other factors have since been shown to induce its expression, including nutrient depletion and calcium disequilibrium [41]. We had previously confirmed that CHOP over-expression promotes apoptosis in renal epithelial cells, and genetic knock out of CHOP protected against ER stress mediated-AKI [7]. In this study, knock out of TDAG51 resulted in reduced CHOP transcription and protein expression in mouse kidneys, suggesting that TDAG51 is an upstream mediator of CHOP, particularly in the tubular epithelium, and induces apoptosis in a CHOP-dependent mechanism. It should be noted that TDKO did not fully inhibit CHOP expression; this is likely due to the fact that CHOP can be stimulated through multiple pathways, which may be induced simultaneously. These pathways include the PERK and IRE1 pathways that induce TDAG51 expression [35, 41], as well as the ATF6 UPR pathway and other ER stress-independent pathways [41, 42]. Interestingly, while ER stress was activated in CKO mice treated with TM (as shown by increased GRP78 expression), apoptosis was prevented. CKO mice with TM-mediated AKI do not exhibit renal injury [7], suggesting kidney damage may not be dependent on UPR activation, but rather on CHOP-induced apoptosis.

In addition to inducing apoptosis, ER stress can also lead to fibrosis. When proteins are unable to properly form their tertiary structure, they cannot function correctly; this can lead to cell shape change [43, 44] if junctional proteins are retained in the ER [45]. Cell shape change can lead to epithelial junctional proteins being reduced (E-cadherin) or translocated (β-catenin), preventing adherens junction formation [45, 46]. Inhibiting ER stress can prevent the expression of fibrotic markers in vitro, as well as fibrosis in vivo [11, 16, 43]; further, genetic knock out of CHOP can protect against renal fibrosis [11, 47]. As mentioned, ER stress can influence fibrosis, and spliced XBP1 is increased in the thick ascending limb of the nephron in a genetic mouse model of inherited renal fibrosis [48]. Notably, spliced XBP1 was significantly reduced in our CKO mouse model of CKD, which did not develop fibrosis [11]. Further, XBP1 (spliced and unspliced) induces collagen production through an autophagic mechanism in hepatic stellate cells [49]. We have previously demonstrated a role for TDAG51 in the development of fibrosis, whereby TDKO mice did not develop TGF-β1-mediated peritoneal fibrosis, though a specific mechanism was not determined [16]. In our current CKD model, TDKO mice demonstrated reduced spliced XBP1 in the kidney. Further, TGF-β1-mediated collagen production is reduced in renal fibroblasts treated with an IRE1 endonuclease inhibitor, demonstrating that TGF-β1-mediated collagen production requires IRE1 activation. Thus, it is likely that the reduced renal interstitial fibrosis found in TDKO mice is due to a TGFβ-IRE1-XBP1 mechanism.

The major pro-fibrotic cytokine responsible for mediating fibrosis is TGF-β1. TGF-β1 is induced by a number of factors, including ER stress [16]. Further, we previously found increased mRNA levels of TGF-β1 and TGF-β receptor 1 (activin receptor-like kinase 5; ALK5) in our mouse model of CKD, and expression of both was reduced in mice given a protein-folding chaperone to inhibit ER stress [11]. TGF-β1 is responsible for inducing TGF-β receptor mRNA [50], as well as protein expression on the cell surface [51]. Increased expression of TGF-β receptors is associated with excessive production of collagen [52]. TGF-β signaling is dependent on its receptors and can be prevented by inactivation/dephosphorylation of TGF-β receptor 1. TGF-β receptor 1 can be inactivated by Smad7 binding to the GADD34/PP1c complex [53]. Notably, we found a significant inhibition of TGF-β receptor 1 in TDKO mice. Further, inhibiting TGF-β receptor 1 prevented splicing of XBP1 in renal epithelial cells. These data suggest that the reduced splicing of XBP1 found in TDKO mice is due to decreased levels of TGF-β receptor 1. In support, TDKO mice given a TGFβ- adenovirus did not develop peritoneal fibrosis [16], indicating that the lack of fibrosis in TDKO mice is not due to reduced levels of TGF-β, but rather a downstream mediator (TGF-β receptor 1). This suggests that TDAG51 may act as a translational regulator of TGF-β receptor 1; TGF-β signaling induces collagen production, causing a greater demand on protein folding and subsequent phosphorylation of IRE1 and XBP1 splicing. Interestingly, platelet-derived growth factor (PDGF) signaling activates the UPR [54, 55] and upregulates the expression of TGF-β receptors 1 and 2 [56]. PDGF also activates additional signaling pathways, including the MEK/ERK pathway [57], which has been implicated as a possible upstream mediator of TDAG51 [58].

**Fig. 7:**
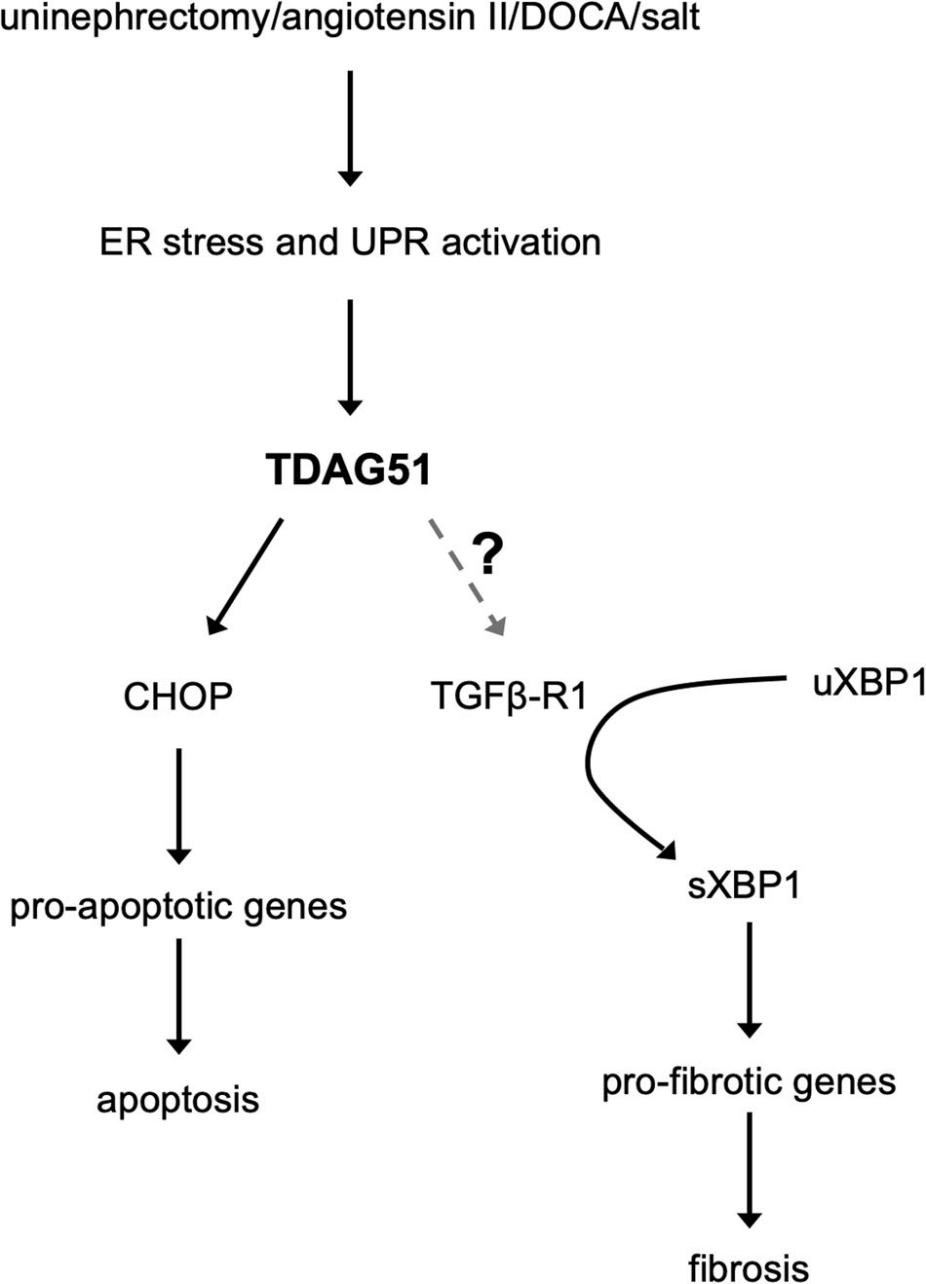
Model for TDAG51 mediated apoptosis (CHOP pathway) and fibrosis (TGF-β receptor 1 and sXBP1 pathway). The induction of CKD, caused by reduced renal mass, increased angiotensin II and aldosterone, and increased salt consumption, triggers endoplasmic reticulum (ER) stress and activation of the unfolded protein response (UPR). This amplifies TDAG51 expression in the kidney. The elevated levels of TDAG51 triggers a pro-apoptotic signaling pathway and a pro-fibrotic signaling pathway. In the pro-apoptotic pathway, TDAG51 induces expression of CHOP, which activates pro-apoptotic genes and, eventually, programmed cell death. In the pro-fibrotic pathway, TDAG51 increases expression of TGF-β receptor 1, which leads to the splicing of XBP1. Spliced XBP1 leads to the activation of pro-fibrotic genes, and the development of renal interstitial fibrosis.

In summary, we have demonstrated that TDAG51 protects against renal cell apoptosis and renal interstitial fibrosis in CKD, through a mechanism independent from blood pressure, albuminuria, and structural damage of the glomeruli. It exerts pro-apoptotic signaling through the ER stress protein CHOP and activates pro-fibrotic signaling through TGF-β receptor 1 and subsequent downstream splicing of XBP1 (Fig. 7). Additional research is required to fully elucidate the novel relationship between TDAG51 and its downstream target TGF-β receptor 1, which will aid in discerning the specific mechanisms responsible for the development and progression of renal interstitial fibrosis in CKD.

## Supplementary information

supplemental material table of contents

Supplemental Figures

## Data Availability

The datasets supporting the conclusion of the article are included within the article.
