# Supplementary material for: TDAG51 induces renal interstitial fibrosis through modulation of TGF-β receptor 1 in chronic kidney disease
Source: Cell Death Dis. 2021 Oct 8;12(10):921. doi: 10.1038/s41419-021-04197-3 (PMC8501078; doi:10.1038/s41419-021-04197-3)
Supplement: Supplementary file 1 — supplemental material table of contents [file 41419_2021_4197_MOESM1_ESM.pdf]

SUPPLEMENTAL MATERIAL TABLE OF CONTENTS

Supplemental Figure 1: Wild type and TDAG51 knockout mouse genotyping.

Supplemental Figure 2: Serum creatinine levels of tunicamycin-treated wild type and TDAG51 knockout mice.

Supplemental Figure 3: HK-2 transfection efficiency.
