# Supplementary figures and images for: TDAG51 induces renal interstitial fibrosis through modulation of TGF-β receptor 1 in chronic kidney disease
Source: Cell Death Dis. 2021 Oct 8;12(10):921. doi: 10.1038/s41419-021-04197-3 (PMC8501078; doi:10.1038/s41419-021-04197-3)

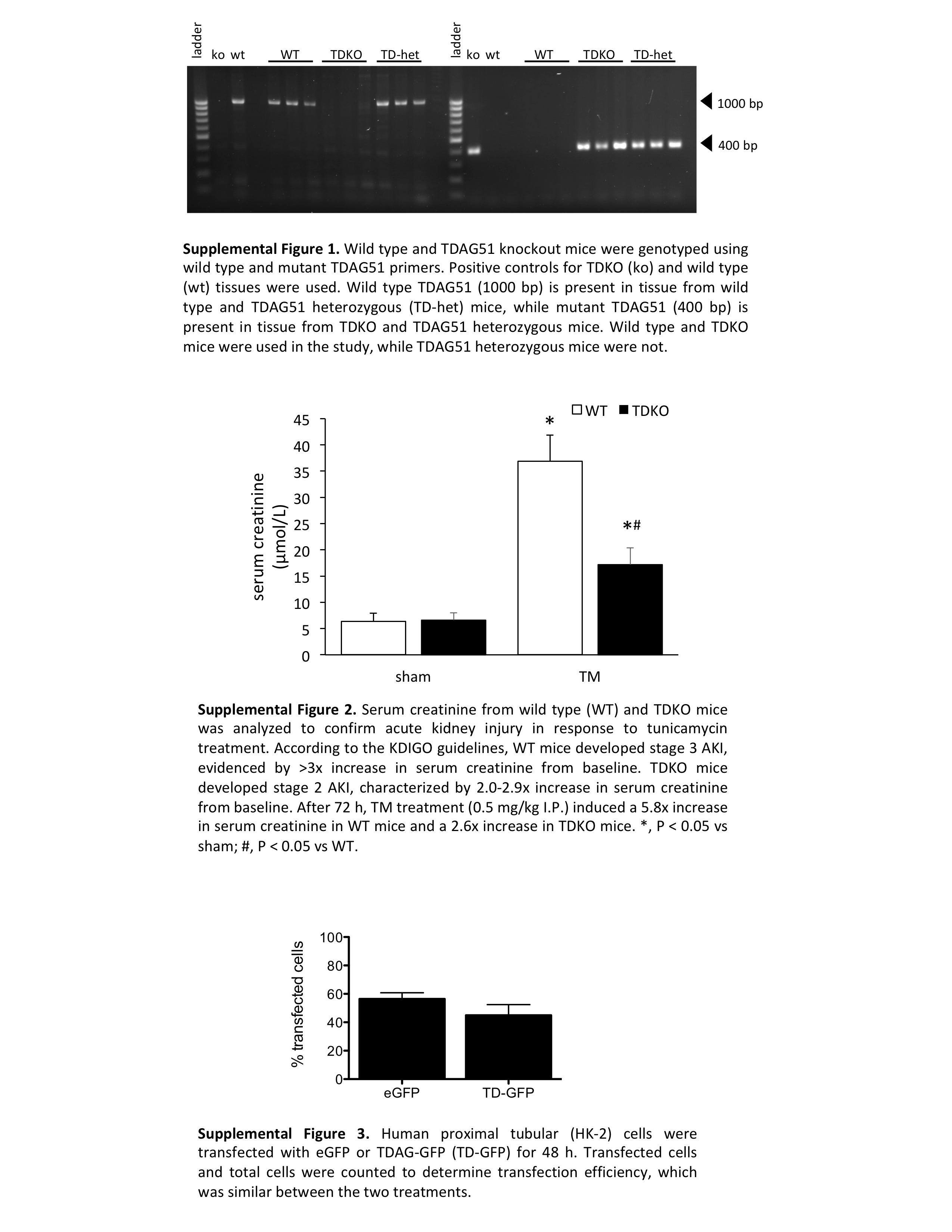

Supplement: Supplementary file 2 — Supplemental Figures [file 41419_2021_4197_MOESM2_ESM.tif]
